# Identification of Ser465 as a novel PINK1 autophosphorylation site

**DOI:** 10.1186/s40035-017-0103-7

**Published:** 2017-12-14

**Authors:** Ji-feng Guo, Ling-yan Yao, Qi-ying Sun, Yi-ting Cui, Yang Yang, Qian Xu, Xin-xiang Yan, Bei-sha Tang

**Affiliations:** 1 Department of Neurology, Xiangya Hospital, Central South University, Changsha, Hunan 410008 People’s Republic of China; 2 Laboratory of Medical Genetics, Central South University Changsha, Changsha, Hunan 410078 China; 3 National Clinical Research Center for Geriatric Disorders, Changsha, Hunan 410078 China; 4 Key Laboratory of Hunan Province in Neurodegenerative Disorders, Central South University, Changsha, Hunan 410008 China; 5 Parkinson’s Disease Center of Beijing Institute for Brain Disorders, Beijing, 100069 China; 6 Collaborative Innovation Center for Brain Science, Shanghai, 200032 China; 7 Collaborative Innovation Center for Genetics and Development, Shanghai, 200433 China

**Keywords:** Parkinson’s disease, PINK1, Autophosphorylation sites, Kinase activity

## Abstract

**Background:**

PINK1 (PTEN-induced putative kinase 1) gene is the causal gene for recessive familial type 6 of Parkinson’s disease (PARK6), which is an early-onset autosomal recessive inherited neurodegenerative disease. PINK1 has been reported to exert both autophosphorylation and phosphorylation activity, affecting cell damage under stress and other physiological responses. However, there has been no report on the identification of PINK1 autophosphorylation sites and their physiological functions.

**Methods:**

(1) We adopted mass spectrometry assay to identify the autophosphorylation site of PINK1, and autoradiography assay was further conducted to confirm this result. (2) Kinase activity assay was used to compare the kinase activity of both Ser465 mutant PINK1 and disease-causing mutant PINK1. (3) We use Pulse-chase analysis to measure whether Ser465 may affect PINK1 degradation. (4) Immunocytochemistry staining was used to study the PINK1 subcellular localization and Parkin transition in subcellular level.

**Result:**

In our study, we identified the 465th serine residue (Ser465) as one of the autophosphorylation sites in PINK1 protein. The inactivation of Ser465 can decrease the kinase activity of PINK1. Either dissipated or excessive Ser465 site phosphorylation of PINK1 can slow down its degradation. PINK1 autophosphorylation contributes to the transit of Parkin to mitochondria, and has no effect on its subcellular localization. PARK6 causal mutations, T313 M and R492X, display the same characteristics as Ser465A mutation PINK1 protein, such as decreasing PINK1 kinase activity and affecting its interaction with Parkin.

**Conclusion:**

Ser465 was identified as one of the autophosphorylation sites of PINK1, which affected PINK1 kinase activity. In addition, Ser465 is involved in the degradation of PINK1 and the transit of Parkin to mitochondria. T313 M and R492X, two novel PARK6 mutations on Thr313 and Arg492, were similar to Ser465 mutation, including decreasing PINK1 phosphorylation activity and Parkin subcellular localization.

## Background

Parkinson’s disease (PD) is one of the most pervasive neurodegenerative diseases. Currently, the etiology and mechanism of PD are not fully clear. But, aging, environmental and genetic factors appear to play the key role in PD [2, 7, 21].

*PINK1* (*PTEN-induced putative kinase 1*) gene is the causative gene for PARK6, which was cloned in 2004 by Valente et al. [20]. Encoded by the *PINK1* gene, PINK1 protein contains 581 amino acids, including a mitochondrial localization domain (34 amino acids) and a serine/threonine kinase domain (354 amino acids). The kinase domain (KD) is highly conserved, capable of catalyzing by binding to the peptide substrate and transferring ATP phosphate group [20]. Recent studies showed that PINK1 may directly phosphorylate other proteins, exerting anti-oxidative stress and anti-apoptosis effects. TRAP1 protein is found to be the substrate of PINK1 [14], through which, PINK1 can reduce the release of mitochondrial cytochrome c and fill the gap caused by cell damage and even cell death under stress. In 2008, Kim et al. [8] found that PINK1 can phosphorylate Parkin directly, regulating their transfer to the mitochondria. In addition, PINK1 kinase domain possesses both autophosphorylation [11] and phosphorylation activity in vitro, such as phosphorylating histone H1 or casein [17, 18]. In our previous work, we found two novel *PINK1* homozygous disease-causing mutations (T313 M, R492X) [6], both located in the kinase domain. Matenia et al. [9] found that microtubule affinity regulating kinase 2 (MARK2) can phosphorylate PINK1, and Thr313 was proved to be the phosphorylation site. When T313 M mutation occurs, PINK1 cannot be phosphorylated, leading to mitochondrial toxicity and abnormal distribution in nerve cells. Arg492 is located at the junction of PINK1 protein kinase domain and the carboxyl terminus. R492X mutation can cause the loss of the entire carboxyl terminal, altering PINK1 kinase activity [6, 17].

There has been no report on the identification of PINK1 autophosphorylation sites in vitro studies and the effect on physiological functions brought by the changes of these sites. Our study demonstrated that Ser465 was one of the autophosphorylation sites in PINK1. The autophosphorylation of PINK1 protein can affect its kinase activity, protein stability, as well as the interaction with Parkin. *PINK1* causative mutations T313 M and R492X may cause PD by affecting its kinase activity and interaction with Parkin.

## Methods

### Plasmid construction

293A human embryonic kidney cells, pGEX-5X-1 vector, pKH3-PINK1 and EGFP-Parkin plasmid were provided by the school of Life Science of University of Science and Technology in China. pKH3-PINK1-T313 M, pKH3-PINK1-R492X and pGEX-5X-1-PINK1 plasmids were constructed in our previous work [3, 22]. pGEX-5X-1-PINK1-T313 M, pGEX-5X-1-PINK1-R492X, pGEX-5X-1-PINK1-S465A, pGEX-5X-1-PINK1-S465D, pKH3-PINK1-S465A and pKH3-PINK1- S465D plasmids were all constructed by this study.

### Protein expression and purification in *E. coli*

We used the same method to induce prokaryotic plasmid expression and purify protein as described in a previous study [3, 22]. A small amount of GST-PINK1 bacteria (112–581 aa) were inoculated into a 600 mL culture medium (AMP+), incubated at 37 °C, 250 rpm. When OD values reached between 0.4–0.6, 100 μl IPTG was added to the culture and incubated for 3 h under the same condition. Resuspended the culture with 1XPBS, followed by sonication, then a crude supernatant was obtained. The protein extract was purified with G4B purification column. SDS-PAGE was used to identify the purified protein, followed by Coomassie brilliant blue staining.

### Kinase assay and mass spectrometry

Autophosphorylation of recombinant PINK1 protein was performed in the reaction system containing 2.4 μL of 10X kinase reaction buffer, 19.6 μL of GST-PINK1 purified protein supernatant, 2 μL of 10 mM ATP. Then the mixture was incubated in 30 °C water bath for 1.5 h. All the phosphorylated proteins were denatured by 5XSDS, heating at 100 °C for 5 min, resolved by SDS-PAGE, followed by brilliant coomassie blue R250 staining for 30 min and decolorizing for 5 min. Excised gel slices containing desired bands, can be stored at 4 °C and sent for mass spectrometry analysis.

### Autoradiography

The proteins were purified by the same method described before. 2.4 μL of 10X kinase reaction buffer, 19.6 μL of purified protein supernatant, 10 μCi of [γ^-32P^] ATP were added, and incubated in 30 °C water bath for 30 min. Then 5XSDS was added, and boiled at 100 °C for 5 min. SDS-PAGE was carried out as described previously, and protein bands were stained with brilliant coomassie blue R250 for 5 min, and decolorized for another 5 min. The plastic wrapped gel and a film in the X-ray film cassette were placed in a dark room. The film was exposed at −80 °C for 8-16 h, followed by developing and fixing.

### Cell culture and transfection

The 293A cells were maintained in the medium containing 10% fetal bovine serum, 100 U/ml penicillin and 100 U/ml streptomycin, and incubated at 37 °C in a CO_2_ incubator. Plasmid transfection was performed according to the Lipofectamine®2000 protocol.

### Immunocytochemistry staining

The immunocytochemical staining of eukaryotic plasmid cells was conducted with the method used in previous articles [3, 22]. The cultured cells were fixed with 4% paraformaldehyde. 0.25% TritonX-100/PBS was used for permeabilization. The samples were blocked in 1% FBS/PBS, and incubated with primary antibody and Rhodamine fluorophores conjugated secondary antibodies. Counterstaining with DAPI, the fluorescence was observed under the inverted fluorescence microscope, then images were captured.

### Pulse-chase analysis

After cell transfection for 24-36 h, each well was added with 100 μg/mL CHX (cycloheximide, CHX). Cells were collected at 0 h, 1 h, 2 h and 3.5 h after the addition of CHX. Total protein was extracted for Western analysis to detect the degradation of the interested protein.

### Statistical analysis

All statistical data were calculated and analyzed using SPSS Statistics 13.0 software. All error bars were expressed as mean ± s.d.. The statistical significance of differences was evaluated by one-way ANOVA, followed by student’s *t*-test.

## Results

### Identification of PINK1 protein autophosphorylation site(s) with mass spectrometry

**Fig. 1:**
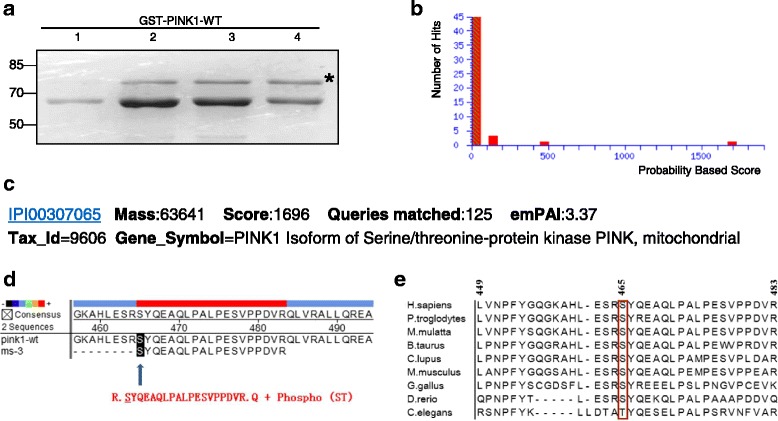
Identification of PINK1 protein autophosphorylation site(s) with mass spectrometry. **a** Coomassie blue staining of purified GST-PINK1-WT fusion protein. The asterisk indicates the band of PINK1, 77 kDa, equivalent of the molecular weight of GST-PINK1-WT fusion protein. **b** Protein score histogram in Mascot with the mass spectrometry data. The horizontal axis stands for protein match score (a higher score indicating a more reliable result), and the vertical axis represents the number of matched protein. The red bar, located around 1600–1700 at the horizontal axis, suggests the autophosphorylation site. **c** Detailed information of the identified autophosphorylation site. **d** A comparison of the identified sequence with the PINK1 protein sequence (NCBI database). The modified serine site (marked by black square), referring to the autophosphorylation site, is located at the 465th serine site in PINK1. **e** Ser465 (in red) displays highly conserved in nine organisms

We firstly constructed GST-PINK1 plasmid and purified the GST-PINK1 protein in large quantities. The purity of the recombinant protein was verified by SDS-PAGE and followed by coomassie blue staining, as shown in Fig. 1a. To identify the autophosphorylation sites of PINK1, we adopted the well-utilized kinase assay with the purified GST-PINK1 protein as both the kinase and substrate. Afterwards, the phosphorylated mix was separated by SDS-PAGE, and the matched bands were excised for mass spectrometry (provided by Beijing Proteome Research Center) and analyzed with Mascot (UK Matrix Science Co.) (Fig. 1b, c, d). The MS/MS data suggested that the auto-phosphorylation site was located in the 465th amino acid in PINK1, which was demonstrated to be well conserved by the homology detection among nine different species (Fig. 1e).

### PINK1 protein autophosphorylation level can be weakened by silencing S465 phosphorylation site

**Fig. 2:**
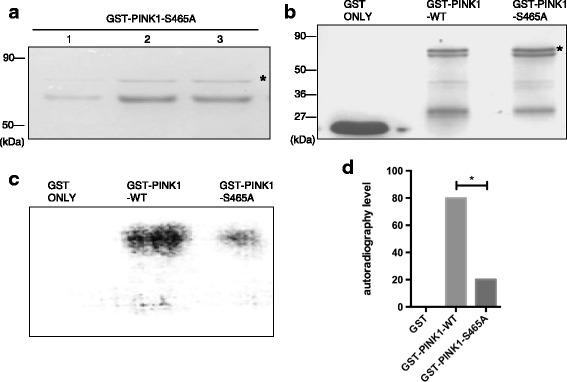
PINK1 protein autophosphorylation can be weakened by silencing S465 phosphorylation site. **a** Coomassie blue staining of purified GST-PINK1-S465A fusion protein. **b** Coomassie blue staining of three kinase phosphorylation reaction systems (GST, GST-PINK1-WT and GST-PINK1-S465A). GST protein band was shown in lane 1, as a negative control. Visible GST-PINK1-WT and GST-PINK1-S465A fusion protein bands were shown in Lane 2 and lane 3 respectively, as shown by asterisk. **c** Autoradiography results of three groups. Two positive bands were shown in lane 2 and 3, which stands for the autophosphorylation level of the two groups with GST-PINK1-WT and GST-PINK1-S465A as the kinases, respectively. No positive bands in lane 1. **d** Quantification of the autophosphorylation activity of GST-PINK1-WT and GST-PINK1-S465A protein based on the result of autoradiography. Compared to the WT fusion protein, the autophosphorylation level of mutant GST-PINK1-S465A protein was lower. *P < 0.05, statistically significant

To further confirm whether Ser465 in PINK1 was the autophosphorylation site, we firstly constructed prokaryotic expression vector pGEX-5X-1-PINK1-S465A, in which serine residue (S) was replaced by alanine residue (A) by site-directed mutagenesis, which cannot be phosphorylated. The GST-PINK1-S465A can be expressed in *E. coli* after induction (Fig. 2a). To detect the change of autophosphorylation level of PINK1 protein after Ser465 was silenced, we performed the kinase phosphorylation reaction with the purified fusion proteins as both kinase and substrate meanwhile. With the involvement of [γ^-32P^] ATP, we can quantify the kinase activity. The experiment was divided into three groups with purified GST, GST-PINK1-WT, GST-PINK1-S465A fusion protein, respectively. The SDS-PAGE was performed after the kinase reaction (Fig. 2b). Autoradiography results were showed in Fig. 2c. No positive bands appeared in the GST only group, which is a negative control; positive bands appeared in both GST-PINK1-WT and GST-PINK1-S465A groups. Compared to the GST-PINK1-WT, the autophosphorylation level of GST-PINK1-S465A was obviously lower (Fig. 2d), implying that the total phosphorylation level was significantly decreased after Ser465 kinase activity was disabled. The result strongly demonstrated that Ser465 was the autophosphorylation site in PINK1. Even though Ser465 was disabled, there were still some detectable weak signals in the GST-PINK1-S465A group, hinting the existence of other autophosphorylation sites besides Ser465 in PINK1.

### Both disease-causing mutant PINK1 protein and Ser465A mutant PINK1 protein decrease its kinase activity

**Fig. 3:**
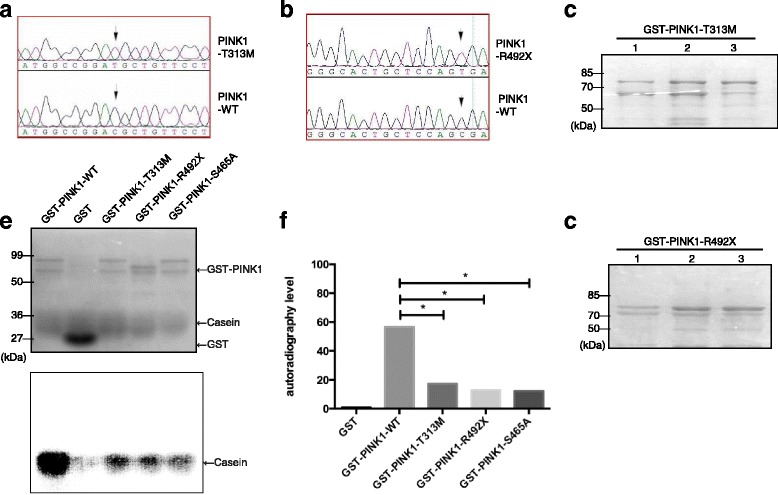
Both disease-causing mutant PINK1 protein and Ser465A mutant PINK1 protein decreased its kinase activity. **a** Sanger sequencing of T313 M mutant PINK1 and WT PINK1. The arrow indicates that the 938th base mutated from C to T, leading to threonine mutated to methionine. **b** Sanger sequencing of R492X mutant PINK1 and WT PINK1. The arrow indicates that the 1474th base mutated from C to T, leading to arginine mutated to a stop code. (**c, d**) Coomassie blue staining of purified GST-PINK1-T313 M and GST-PINK1-R492X fusion proteins. **e** Wild-type and three mutant GST-PINK1 proteins phosphorylation activity with casein as the common substrate. The upper one is the result of Coomassie Blue staining. Each lane represents GST-PINK1-WT fusion protein, GST protein, GST-PINK1-T313 M fusion protein and GST-PINK1-R492X fusion protein, respectively. The lower one was the result of autoradiography of five groups. Except GST group, four positive signals appeared in the other four groups, standing for the casein phosphorylated by GST-PINK1-WT fusion protein, GST-PINK1-T313 M fusion protein, GST-PINK1-R492X fusion protein and GST-PINK1-S465A fusion protein respectively. **f** Bar graph showing the quantification of phosphorylation activity of the five groups. The difference between three mutant PINK1 proteins and WT protein were statistically significant (^*^ *P* < 0.05)

In our previous work, we found two novel *PINK1* homozygous disease-causing mutations, T313 M and R492X, which are mutations on Thr313 and Arg492, respectively. As the two sites are both kinase domain located, they may cause disease by affecting PINK1 kinase activity. In order to investigate whether T313 M and R492X mutations affect its kinase activity, we constructed prokaryotic expression vector of T313 M and R492X PINK1 plasmid, pGEX-5X-1-PINK1-T313 M and pGEX-5X-1-PINK1-R492X (Fig. 3a, b), which were purified as described above (Fig. 3c, d). Ser465A is also the mutant PINK1 protein, which can disable the autophosphorylation activity of Ser465 in PINK1, as described above. Five phosphorylation reaction groups were designed with casein as the common substrate and the fusion proteins as the kinases, respectively, including GST protein, GST-PINK1-WT protein, GST-PINK1-T313 M protein, GST-PINK1-R492X, GST -PINK1-S465A protein. After the phosphorylation reaction, SDS-PAGE was performed (Fig. 3e). The autoradiography results were shown in Fig. 3e. No positive bands appeared in GST only group. The levels of phosphorylated casein were decreased in the three mutant protein groups, GST-PINK1-T313 M, GST-PINK1-R492X and GST-PINK1-S465A, compared with GST-PINK1-WT group. The differences between three mutants and the wild-type PINK1 protein were statistically significant (*P* < 0.05), respectively (Fig. 3f).

### S465 has no influence on PINK1 subcellular localization

**Fig. 4:**
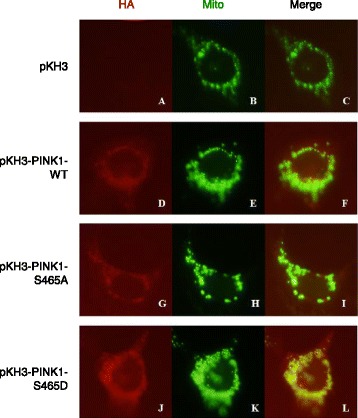
S465 has no influence on PINK1 subcellular localization. **a**, **b**, **c**, the subcellular localization in HEK 293 cells transfected with pKH3, as negative control; (**d**, **e**, **f**), the subcellular localization in HEK 293 cells transfected with pKH3-PINK1-WT; (**g**, **h**, **i**), the subcellular localization of pKH3-PINK1-S465A transfected cells; (**j**, **k**, **l**), the subcellular localization of pKH3-PINK1-S465D transfected cells. In A, D, G and J, the red fluorescence illustrated the subcellular localization of proteins containing the HA tag; In B, E, H and K, the green fluorescence stood for mitochondria; In C, F, I and L, co-localization (*in yellow*) of both HA-tag containing protein (*in red*) and mitochondria (*in green*)

In order to further clarify the meaning of Ser465 autophosphorylation in eukaryotic cells, we constructed prokaryotic expression vector of S465A and S465D mutant PINK1, pKH3-PINK1-S465A and pKH3-PINK1-S465D. S465A was described as above, which cannot be phosphorylated. In opposite, aspartic acid residues (D) took the place of the serine residue (S), which was phosphorylation-mimic. We then transfected pKH3-PINK1-WT, pKH3-PINK1-S465A, pKH3- PINK1-S465D in HEK293 cells respectively, with the pKH3 vector as a blank control, followed by immunofluorescence staining. The results showed that pKH3-PINK1-WT protein was located in the cytoplasm, punctate distribution, co-localized with the Mito staining, showing a typical mitochondrial localization (Fig. 4f); No significant change of subcellular location for the two mutant PINK1 protein, pKH3-PINK1-S465A and pKH3-PINK1-S465D, still displaying the same co-localization with mitochondria in the cytoplasm (Fig. 4i, l).

### S465 phosphorylation site affects PINK1 degradation

**Fig. 5:**
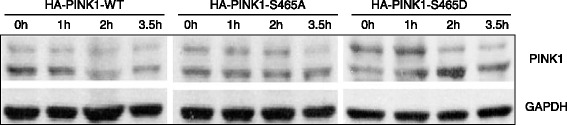
S465 phosphorylation site affects PINK1 degradation. The first four lanes represented the quantity of HA-PINK1-WT protein in HEK293 cells transfected with pKH3-PINK1-WT plasmid, collected 0 h,1 h, 2, 3.5 h after the addition of actidione. Similarly, the middle and last four lanes represented HA-PINK1-S465A and HA-PINK1-S465D protein, respectively

HA-PINK1-WT, HA-PINK1-S465A and HA-PINK1-S465D plasmids were transfected into HEK293 cells, followed by pulse chase experiment to measure protein degradation. The results showed that degradation half-life time of WT PINK1 protein was about 2 h; For the HA-PINK1-S465A protein and HA-PINK1-S465D protein, the degradation half-life time of both were about 3.5 h, slower than the WT PINK1 protein (Fig. 5). The phosphorylation of S465 may be related to the degradation of PINK1, as both disabled phosphorylation and excessive phosphorylation of Ser465 increase the half-life time and the concentration of PINK1 in cells. The normal phosphorylation of Ser465 may be required to maintain a stable level of PINK1 and cell homeostasis.

### Both disease-causing mutant PINK1 protein and Ser465A mutant PINK1 protein affect Parkin subcellular localization

**Fig. 6:**
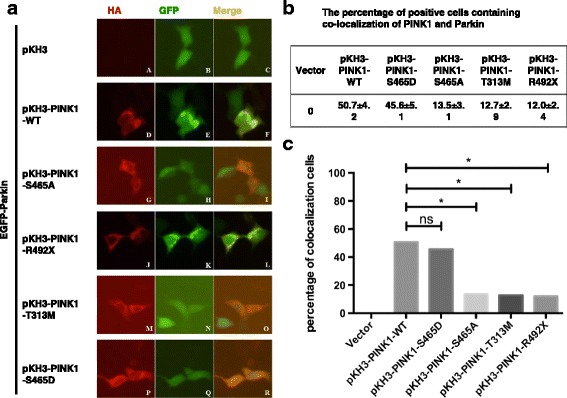
Both disease-causing mutant PINK1 protein and Ser465A mutant PINK1 protein affects Parkin subcellular localization. **a** The six rows represented the subcellular localization of co-transfection of EGFP-Parkin with pKH3, pKH3-PINK1-WT, pKH3-PINK1-S465A, pKH3-PINK1-S465D, pKH3-PINK1-T313 M and pKH3-PINK1-R492X, respectively. The red fluorescence showed the subcellular localization of PINK1 protein containing HA tag in the first column; The green fluorescence illustrated subcellular localization of Parkin protein containing GFP tag in the second column; The yellow fluorescent showed the co-localization of both PINK1 (*red*) and Parkin (*green*) in the third column. **b** The percentage of positive cells containing colocalization of PINK1 and Parkin in six co-transfection groups of EGFP-Parkin with pKH3 vector, pKH3-PINK1-WT, pKH3-PINK1-S465A, pKH3-PINK1- S465D, pKH3-PINK1-T313 M and pKH3-PINK1-R492X. **c** Quantification of the percentage of PINK1/Parkin colocalization cell. (*P < 0.05, statistically significant)

Studies have shown that PINK1 can phosphorylate Parkin protein directly, and translocate Parkin to the mitochondria, which gathers in clusters around the nucleus [10]. It is still unclear whether S465 autophosphorylation site can affect the subcellular localization of Parkin. In our study, we found that EGFP-Parkin protein evenly distributed in the cytoplasm when cells were transfected with pKH3-EGFP-Parkin only (Fig. 6a); when cells were co-transfected EGFP-Parkin with pKH3-PINK1-WT or pKH3-PINK1-S465D, colocalization of Parkin with PINK1 in clustered mitochondrias was observed (Fig. 6a); when cells were co-transfected EGFP-Parkin with mutant PINK1, pKH3-PINK1-S465A, pKH3-PINK1-T313 M or pKH3-PINK1-R492X, respectively, EGFP-Parkin evenly distributed in the cytoplasm (Fig. 6a), and each mutant PINK1 protein exhibited scattered distribution in cytoplasm, was located in mitochondria (Fig. 6a). The percentage of cells containing colocalization of PINK1 with Parkin protein was shown in Fig. 6b. We compared the four mutant PINK1 co-transfected with EGFP-Parkin groups and the WT PINK1 group, three of them (pKH3-PINK1-S465A, pKH3-PINK1-T313 M and pKH3- PINK1-R492X) exhibited obvious decreased percentage of positive cells compared with WT PINK1 (*P < 0.05), and the difference was not statistically significant (*P > 0.05) in pKH3-PINK1-S465D group compared with the WT group (Fig. 6c).

## Discussion

The dynamic changes of protein play a key role in life, as they can achieve the physiological function through various post-translational modifications, among which, reversible protein phosphorylation is a common and crucial one. In mammals, there are about 30% of the proteins going on with phosphorylation process. A variety of biological processes are closely related to post-translational protein phosphorylation, which has been playing an on/off regulation in numerous biochemical functions, such as transcriptional regulation, signal transduction, DNA repair, apoptosis regulation. Therefore, the phosphorylation of proteins is an essential part in intracellular signaling transduction.

In our experiment, we used in vitro phosphorylation studies to exclude the possibilities of PINK1 phosphorylated by other proteins, adopted a liquid chromatography mass spectrometry to identify the Ser465 site as the PINK1 protein autophosphorylation site. Since phosphorylation is a reversible process, the loss of phosphate groups was possible under the experimental conditions. In addition, the phosphorylated peptides were relatively little compared with non-phosphorylated peptides, which could be concealed easily when identified in the mass spectrometry, leading to the false negative results. Therefore, besides Ser465 site, there may be other unidentified PINK1 autophosphorylation sites.

In order to identify whether Ser465 was the PINK1 autophosphorylation site or not, we firstly constructed Ser465 autophosphorylation silent type pGEX-5X-1-PINK1-S465A, replacing the serine residue (S) with alanine residue (A) which cannot be phosphorylated, using the site-directed mutagenesis. After Ser465 being silenced, the PINK1 protein autophosphorylation level was decreased, but not completely blocked, indicating that Ser465 is just one of the PINK1 autophosphorylation sites.

Our study found that the inactivation of Ser465 can down-regulate the kinase activity of PINK1. Together with Ser465A, T313 M and R492X mutant PINK1 also decrease kinase activity in vitro, both of which are disease-causing mutations of PINK1, implying that decreased kinase activity may contribute to PD. However, experiments in vitro cannot fully simulate the phosphorylation process of eukaryotic cells in vivo, and the specific mechanism remains to be further studied.

When we transfected two pKH3-PINK1 mutants in HEK293 cells, HA-PINK1-S465A and HA-PINK1-S465D, there were no significant changes of subcellular localization of PINK1, still displaying the mitochondrial localization. These results suggest that the Ser465 site has no effect on PINK1 subcellular localization. PINK1 protein is an integral membrane protein, mainly located in the mitochondria. It has already shown that PINK1 protein localizes in mitochondria cristae, and both wild-type and mutant (outside the mitochondrial localization domain) PINK1 protein distribute in the same way in cells, indicating that PINK1 mutation outside the mitochondrial localization domain does not affect its subcellular localization [12]. As a mitochondrial-located kinase, PINK1 plays a key role in maintaining the normal function of mitochondria and protecting the mitochondria from oxidative stress.

Our study found that Ser465 phosphorylation was also related to the PINK1 degradation. Both disabled or excessive PINK1 phosphorylation can slow down the PINK1 degradation, increase the intracellular concentration of PINK1. A proper concentration of PINK1 is required to maintain mitochondrial homeostasis. Most studies have found that PINK1 mainly maintains normal mitochondrial morphology and function, reduces mitochondrial dysfunction under stress, inhibits apoptosis, and is a neuron protective protein ([1, 4, 5, 13, 15]). Some studies have demonstrated that PINK1 protein degrades primarily by the ubiquitin-proteasome degradation pathway, but so far the related E3 ligase in the pathway remains unclear [16, 19, 23], which may be associated with the PINK1 autophosphorylation.

Our study also investigated the influence of PINK1 autophosphorylation on Parkin. Both wild-type PINK1 protein and S465D mutant protein can promote the transfer of Parkin to clustered mitochondria, while S465A, T313 M, R492X mutant PINK1 had a relatively weaker role in the interaction with Parkin. S465A mutant can disable the autophosphorylation of PINK1, repressing the transfer process; While mutant S465D possesses the phosphorylation ability, completing the same process as the wild-type protein. We can infer that PINK1 autophosphorylation contributes to the transit of Parkin to mitochondria. For the S465A, T313 M and R492X mutant protein, the inhibition of transfer process may be related to the reduced kinase activity. Some studies^[10]^ have also confirmed that PINK1 can phosphorylate Parkin and jointly transfer to the mitochondria, which were gathered in clusters around the nucleus. The decreased kinase activity of these mutant PINK1 proteins may lead to the abnormal physiological processes.

## Conclusion

In summary, protein phosphorylation is a crucial aspect of intracellular signaling. Our study elucidated that PINK1 protein autophosphorylation can affect its kinase activity and protein stability, and influent PINK1 and Parkin protein interaction. We also included two *PINK1* causative mutations in our study, T313 M and R492X, which can decrease the kinase activity and affect the interaction with Parkin in the same way as Ser465A, hinting that the change of autophosphorylation activity of PINK1 may be one of the mechanisms of the two disease-causing mutations.
